# The prevalence of premature ejaculation and its relationship with polygamous men: a cross-sectional observational study at a tertiary hospital in Somalia

**DOI:** 10.1186/s12894-021-00942-0

**Published:** 2021-12-16

**Authors:** Abdikarim Hussein Mohamed, Hussein Ali Mohamud, Adem Yasar

**Affiliations:** Mogadishu Somalia Turkish Training and Research Hospital, Mogadishu, Somalia

**Keywords:** Premature ejaculation, Polygamy, Erectile dysfunction, Sexual partners

## Abstract

**Background:**

Premature ejaculation (PE) is the most common and prevalent sexual disorder among men. To the best of our knowledge, this is the first study aimed at evaluating the relationship of PE among polygamous men.

**Method:**

Over a 1-year period, a cross-sectional observational study was carried out among 202 married men who visited the urology polyclinic due to different clinical conditions and contributed by completing a standardized structured questionnaire regarding their sociodemographic data, as well as sexual and past medical history.

**Results:**

In our study, the prevalence of PE was 37.1%; half of the monogamous men (50%) complained of PE, while 22% of men with two wives, 20% of men with three wives, and 12% of men with four wives complained of PE (*p* < 0.0001, 95% CI 0.122–1.920). Seventy percent of erectile dysfunction (ED) patients had PE concurrence (*p* < 0.0001, 95% CI 0.057–5.543). Regarding frequency of sexual intercourse, 48% of patients who complained of PE performed sexual intercourse less than two times/week, while two-thirds of the participants who did not complain of PE had sexual intercourse two to four times/week (*p* < 0.0001, 95% CI 0.203–0.568). Among the men who reported ED, 42% had one wife, 21.5% had two wives, 40% had three wives, and 12.5% had four wives (*p* < 0.029, 95% CI 0.417–0.962).

**Conclusions:**

We report that polygamous men have a lower incidence of premature ejaculation and higher sexual satisfaction than monogamous men. There is a significant association between ED and PE, showing a complex and bidirectional relationship between the two conditions. The new taxonomic entity called loss of control of erection and ejaculation (LCEE) views the two sexual symptoms as deeply interrelated. The study results indicate that a sexual intercourse frequency of two or more times per week significantly lowers the risk of PE.

## Background

Premature ejaculation is the most common and prevalent sexual disorder among men, affecting 30% to 50% of the male population [1]. Premature ejaculation is the inability to sufficiently delay ejaculation before or within 60 s of satisfactory sexual intercourse which results in negative sexual effects as defined by the International Society for Sexual Medicine (ISSM) [2]. Waldinger et al. [3] advocated a new classification of premature ejaculation in addition to the two former lifelong PE and acquired PE (APE), i.e., natural variable PE (NVPE) and premature-like ejaculatory dysfunction (PLED).

PE has a significant psychosocial effect that leads to a low quality of life for both partners [4]. Men with PE frequently feel shame and embarrassment with inferiority feelings, dissatisfaction, and feelings of being incapable of satisfying their partner which raises the risk of depression [5].

Tsai et al. [6] reported that erectile dysfunction is the leading risk factor for about 36% to 50% of PE. ED and PE should not be considered to be separate entities because of their complex and bidirectional relationship between the two conditions. Colonnello et al. [7] introduced a new taxonomic entity called loss of control of erection and ejaculation (LCEE), as the first definition of a disorder that views the two sexual symptoms as deeply interrelated, and helps the assessment of concomitant presence of PE and ED, and to improve both PE diagnosis and management. Kempeneers et al. reported that PE decreases with age due to sexual experience and relationship maturation with a partner. Premature ejaculation is associated with anxiety, early and limited sexual practice, poor health status, weak sexual desire, lower frequency of sexual intercourse, diabetes, chronic prostatitis, metabolic syndrome, and drinking habits [8].

Several studies have focused on the impact of premature ejaculation and depression [9] and female sexual satisfaction for men with premature ejaculation [10]. Regarding premature ejaculation among polygamous men (i.e., marrying more than one woman at the same time), no previous studies in the literature have evaluated the impact of polygamy on PE. The main objective of this study was to investigate the relationship between premature ejaculation among polygamous men. In addition, there are no previous studies that have evaluated the prevalence of PE among Somalian men.

## Method

Over a 1-year period, a non-interventional cross-sectional observational study was carried out among married men who visited the urology polyclinic due to different clinical conditions. Men with one or more female sexual wives and having regular sexual intercourse for the last six months were included in the study. Men with diabetes, taking benign prostate hyperplasia (BPH) medications, or who had undergone a prostate operation, and men using antipsychotic drugs were excluded from the study. The diagnostic criterion of premature ejaculation was as defined according to the International Society for Sexual Medicine (ISSM) definition criteria. A total of 202 men were selected for the study and contributed by completing a standardized structured questionnaire that collected data on sociodemographic characteristics (age, number of partners), frequency of sexual intercourse per week and the coexistence of erectile dysfunction, sexual satisfaction, the intravaginal ejaculatory latency time (IELT) reported by the patient, and the premature ejaculation diagnostic tool (PEDT).

The intravaginal ejaculatory latency time (IELT) is the average time between vaginal entrance and ejaculation estimated by the patient. An IELT of < 2 min was considered to be PE. A premature ejaculation diagnostic tool (PEDT) that consisted of five questions was used to assess for all of the participants a diagnosis of premature ejaculation. A score of < 8 equals no PE, a score of 9 or 10 means probable PE, and a score of > 11 indicates PE [4]. The coexistence of erectile dysfunction was measured using the International Index of Erectile Function (IIEF) questionnaire, i.e., a validated questionnaire that contains 15 items (five domains), of which 6 items assess erectile function. A score of < 22 was considered to be the coexistence of ED (LCEE) [11]. To prevent the underestimation of the seriousness of the condition or its effect on the relationship of the partners, absolute PE that is generalized occurring irrespective of partners or relative that is situational or confined to a partner were considered true PE.

This study was approved by the Clinical Research Ethics Committee of the Mogadishu Somali Turkish Training and Research Hospital (approval number MSTH/4410). All methods were performed in accordance with the relevant guidelines and regulations. Participants were informed about the purpose of the study, and written informed consent for participation was obtained from all of the participants.

A univariate descriptive analysis was used for data analysis and expressed as the mean ± standard deviation (SD). The chi-square test and cross-tabulations were also used. A bivariate analysis was used to determine the correlation between premature ejaculation and sexual partners, sexual frequency, age, erectile dysfunction, and sexual satisfaction. The binary logistic regression model used variables that displayed significance in the bivariate analysis and the 95% confidence interval was also calculated. A *p* value of < 0.05 was considered to be statistically significant. The statistical analyses were accomplished using IBM SPSS for Windows version 23.

## Results

In this cross-sectional observational study, there was a total of 202 married men who completed the questionnaire. The mean age of the participants was 39.76 ± 12.04 years and the prevalence of PE was 37.1%. The assessment with a premature ejaculation diagnostic tool (PEDT) revealed that 25% of the participants had PE, while 12% had probable PE, and 63% had no PE.

In this study, the participants were categorized into four age groups, i.e., 20–29 years (21.8%), 30–39 years (34.2%), 40–49 years (21.8%), and over 50 years (22.3%). There was no statistically significant association between age groups and PE (*p* = 0.251, 95% CI 1.443–26.881).

Regarding sexual partners, 56.4% (114 of 202 participants) had one wife, 32.2% (65/202) had two wives, 7.4% (15/202) had three wives, and 4% (8/202) had four wives. Half of the monogamous men (50%) complained of premature ejaculation; among polygamous men, 22% who had two wives, 20% who had three wives, and 12% who had four wives complained of PE. There was a statistically significant correlation between the number of sexual partners and PE (*p* < 0.0001, 95% CI 0.122–1.920) (Table 1).

**Table 1:** Correlation between variables and patients with and without PE

Variables	No. patients (202)	With PE (75)	Without PE (127)	*p* value	95% CI
Age groups	39.76 ± 12.04			0.251	0.066–4.229
20–29	44 (21.8%)	19	25		
30–39	69 (34.2%)	27	42		
40–49	44 (21.8%)	14	30		
> 50	45 (22.3%)	15	30		
Sexual partners	1.58 ± 0.79			< 0.0001	0.122–1.920
1 Wife	114 (56.4%)	57	57		
2 Wife	65 (32.2%)	14	51		
3 Wife	15 (7.4%)	3	12		
4 Wife	8 (4%)	1	7		
Sexual frequency/week	2.64 ± 1.75			< 0.0001	0.298–5.193
≤ 1 time/week	58 (28.7%)	36	22		
2–4 times/week	117 (57.9%)	33	84		
> 4 times/week	27 (13.4%)	6	21		
ED				< 0.0001	0.057–5.543
Yes	69 (34.2%)	48	21		
No	133 (65.8%)	27	106		
Sexual satisfaction				< 0.0001	0.038–5.517
Normal	127 (62.9%)	13	114		
Low	75 (37.1%)	62	13		

A *p* value of < 0.05 was considered to be statistically significant

Regarding the frequency of sexual intercourse per week, 58 (28.7%) men performed sexual intercourse one time or less per week (1, 2, and 3 times per month), 117 men performed sexual intercourse two to four times per week, and 27 men performed sexual intercourse more than four times per week. We noted a statistically significant difference in the number of sexual partners and frequency of sexual intercourse per week (*p* = 0.016) (Table 2). On the one hand, among the participants who complained of PE, 48% (36 participants) performed sexual intercourse one time or less per week, 33 participants performed sexual intercourse from two to four times per week, and only six participants performed sexual intercourse more than four times per week. On the other hand, among the participants without PE, two-thirds (84 participants) performed sexual intercourse from two to four times per week, 21 participants performed sexual intercourse more than four times per week, while 17% performed sexual intercourse one time or less per week. There was a statistically significant correlation between the frequency of sexual intercourse per week and PE (*p* < 0.0001, 95% CI 0.203–0.568).

**Table 2:** Frequency of sexual intercourse per week among sexual partners

Sexual Partners	Sexual frequencies per week			*p* value
	≤ 1time/week	2–4 times/week	> 4 times/week	
1 Wife	41	63	10	*p* < 0.016
2 Wife	14	38	13	
3 Wife	3	8	4	
4 Wife	0	8	0	

A *p* value of < 0.05 was considered to be statistically significant

Thirty-four percent of the participants had ED; 48 out of 69 of the participants with ED had PE concurrence (LCEE), while 36% with PE had no coexistence with ED. A statistically significant association between ED and PE was revealed throughout the study (*p* < 0.0001, 95% CI 0.057–5.543); 42% of men with one wife, 21.5% of men with two wives, 40% of men with three wives, and 12.5% of men with four wives reported ED (*p* < 0.029, 95% CI 0.417–0.962) (Table 3).

**Table 3:** Distribution of erectile dysfunction among sexual partners

Sexual partners	ED		*p* value	95% CI
	Yes	No		
1 Wife	48	66	0.029	0.417–0.962
2 Wife	14	51		
3 Wife	6	9		
4 Wife	1	7		

A *p* value of < 0.05 was considered to be statistically significant

The mean self-reported intravaginal ejaculatory latency time (IELT) was 4.99 ± 2.15 min; IELTs of < 1 min, 1–2 min, and > 2 min accounted for 22.8%, 13.9%, and 63.4%, respectively. There was a significant correlation between sexual partners and IELT (*p* < 0.001) (Table 4).

**Table 4:** Association of intravaginal ejaculatory latency time (IELT) and sexual partners

Sexual partners	IELT			*p* value
	< 1 min	1–2 min	> 2 min	
1 Wife	37	19	58	*p* < 0.0001
2 Wife	6	8	51	
3 Wife	2	1	12	
4 Wife	1	0	7	

A *p* value of < 0.05 was considered to be statistically significant

## Discussion

Due to the lack of a standardized definition, diagnostic tools, and the social hypersensitivity of the disease, the results of studies on the risk factors of PE are still controversial. A multicenter cross-sectional study by Zhang et al. [12] investigated the risk factors of PE and reported that ED, weak sexual desire, lower frequency of sexual intercourse, diabetes, chronic prostatitis, primary married status, lower body mass index, higher age, education level, monthly income, office work, drinking habits, and decreased force of ejaculation were significantly associated with PE. There are no previous studies in the literature that have evaluated the impact of polygamy in PE. Our study indicated that there is a statistically significant difference between the number of sexual partners and PE. Polygamous men have a lower incidence of premature ejaculation and higher sexual satisfaction than monogamous men. Potential reasons include increased sexual experience, relationship maturation, increased sexual intercourse frequency, and psychosexual comfort.

Existing studies regarding the association between age and premature ejaculation are limited. Our study reported no statistically significant association between age groups and PE which was compatible with a multicenter internet-based survey from the Korean Andrological Society in young and middle-aged men that noted no significant differences among PE according to age categories [13]. In contrast to our findings, Kempeneers et al. [8] reported that PE decreased with age due to sexual experience and relationship maturation with a partner.

Sexual frequency is a risk factor of PE and has a clear impact. According to the existing literature, there is no approved frequency of sexual intercourse that lowers or prevents PE. In our study, we acknowledged that a lower frequency of sexual intercourse was significantly related to a higher incidence of PE, which was consistent with a 10-year interval web-based survey on the prevalence and risk factors of PE (The Korean Internet Sexuality Survey (KISS) 2016) by Song, W. H. et al. similarly reported that a low frequency of sexual intercourse per month was related to PE [14]. The study results indicate that a sexual intercourse frequency of two or more times per week significantly lowers the risk of PE.

In our study, the prevalence of PE was 37.1%, which was higher than that reported in previous studies [4, 15, 16]. A study by Karabakan et al. [17], in young Turkish men, revealed a low prevalence of approximately 9.2% PE. The Global Online Sexuality Survey among Arabic Males (GOSS‐AR‐M) published a much higher prevalence of approximately 83.7% PE [18]. A higher PE rate (40.6%) as compared with our study was also reported in a cross‐sectional study conducted at a primary care clinic [19]. The prevalence of PE varies among studies due to the lack of a universally accepted definition and diagnostic tools, as well as the social hypersensitivity of the disease, and the various methods of data collection and analysis.

Several studies have evaluated the association between PE and ED and their co-occurrence, and they have shown a complex relationship between the two diseases. A large, randomized study of 4997 heterosexual men with regular sexual intercourse, aged 18–65 years from nine Asia–Pacific countries, presented that more than 30% of PE patients have concomitant ED, which was in agreement with our findings [20]. Tsai et al. [6] reported that erectile dysfunction is the leading risk factor for about 36% to 50% of PE. A new taxonomic entity called loss of control of erection and ejaculation (LCEE) was introduced by Colonnello et al. [7] that sights the two sexual symptoms as deeply interrelated, and helps the assessment of concomitant presence of PE and ED, and to improve both PE diagnosis and management. A systemic review and meta-analysis of 18 articles, for a total of 57,229 patients, of which 12,144 (21.2%) patients had PE, reported a bidirectional relationship between PE and ED; the presence of PE was associated with a significant increase in ED risk (odds ratio 3.68 (2.61, 5.18), *p* < 0.0001) [21]. Consistent with these studies, in this study, we reported that men with PE have a two-fold risk of developing ED due to anxiety, interpersonal distress, and partner dissatisfaction. In our study, the prevalence of ED was higher in PE patients (64%). Premature ejaculation places a significant burden on an individual and their partner associated with psychological distress and dissatisfaction. Xi et al. reported that when diagnosing erectile dysfunction in patients with PE, SHIM has a sensitivity of about 100% while has a specificity only about 36%; meanwhile, the IIEF-EF is has a higher sensitivity and specificity of about 100% and 77.2% respectively. The authors of this study suggested that the cutoff of SHIM and IIEF-EF should be lowered when assessing erectile function among PE population (SHIM at 17.5 and IIEF-EF at 24.5, respectively). Larger trials are needed to further validate and to expose about the relationship between PE and ED and its association in monogamous and polygamous families. This study mainly focuses the relationship between PE and polygamy compared to monogamous men, and to the best of our knowledge, this is the first study aimed at evaluating the relationship of PE among polygamous men [22].

Neurophysiological, psychosocial, and cognitive factors are some of the mechanisms that involve the pathophysiology of the disease [23]. The coexistence of PE and ED should be carefully evaluated and should not be considered to be separate entities that would increase treatment failure rates [24].

## Strengths and limitations

The limitations of this study include the following: (1) the number of the selected participants is small; however, finding men with more than one wife is challenging; (2) the substantial exclusion criteria, although the study intended to examine the influence of polygamy on PE; (3) the lack of information on women, and the validity of erectile function assessment questionnaires in premature ejaculation patients. Further studies are needed to address the relationship between PE and the position of sexual intercourse (missionary vs. non-missionary), and the age of wives.

## Conclusions

In this study, we report that polygamous men have a lower incidence of premature ejaculation and higher sexual satisfaction than monogamous men. There is a significant association between ED and PE, showing a complex and bidirectional relationship between the two conditions. The new taxonomic entity called loss of control of erection and ejaculation (LCEE) views the two sexual symptoms as deeply interrelated. The study results indicate that a sexual intercourse frequency of two or more times per week significantly lowers the risk of PE.

## Data Availability

Data included in the manuscript.

## Abbreviations

PE: Premature ejaculation
ED: Erectile dysfunction
LPE: Lifelong PE
APE: Acquired PE
NVPE: Natural variable PE
PLED: Premature-like ejaculatory dysfunction
ISSM: International Society for Sexual Medicine
IELT: Intravaginal ejaculatory latency time
PEDT: Premature ejaculation diagnostic tool
IIEF: International Index of Erectile Function
SD: Standard deviation
